# Impact of Dataset Size on 3D CNN Performance in Intracranial Hemorrhage Classification

**DOI:** 10.3390/diagnostics15020216

**Published:** 2025-01-18

**Authors:** Chun-Chao Huang, Hsin-Fan Chiang, Cheng-Chih Hsieh, Bo-Rui Zhu, Wen-Jie Wu, Jin-Siang Shaw

**Affiliations:** 1 Department of Radiology, MacKay Memorial Hospital, Taipei 104, Taiwan; hcc.5306@mmh.org.tw (C.-C.H.); chf.5366@mmh.org.tw (H.-F.C.); sh9102.5307@mmh.org.tw (C.-C.H.); 2 Department of Medicine, MacKay Medical College, New Taipei City 252, Taiwan; 3 Mackay Junior College of Medicine, Nursing, and Management, Taipei 112, Taiwan; 4 Institute of Mechatronic Engineering, National Taipei University of Technology, Taipei 106, Taiwan; a8981030887@gmail.com (B.-R.Z.); t111408017@ntut.org.tw (W.-J.W.)

**Keywords:** intracranial hemorrhage, deep learning, 3D convolutional neural networks, artificial intelligence, CT

## Abstract

**Background:** This study aimed to evaluate the effect of sample size on the development of a three-dimensional convolutional neural network (3DCNN) model for predicting the binary classification of three types of intracranial hemorrhage (ICH): intraparenchymal, subarachnoid, and subdural (IPH, SAH, SDH, respectively). **Methods:** During the training, we compiled all images of each brain computed tomography scan into a single 3D image, which was then fed into the model to classify the presence of ICH. We divided the non-hemorrhage quantities into 20, 30, 40, 50, 100, and 150 and the ICH quantities into 20, 30, 40, and 50. Cross-validation was performed to compute the average area under the curve (AUC) over the last five iterations. The AUC and accuracy were used to evaluate the performance of the models. **Results:** Fifty patients, each with the three ICH types, and 150 non-hemorrhage cases were enrolled. Larger sample sizes achieved stable and acceptable performance in the artificial intelligence (AI) models, whereas training with a limited number of cases posed the risk of falsely high AUC values or accuracy. The overall trends and fluctuations in AUC values were similar between IPH and SDH but different for SAH. The accuracy of the results was relatively consistent among the three ICH types. **Conclusions:** The 3DCNN technique can be used to develop AI models capable of detecting ICH from limited case numbers. However, a minimal case number must be provided. The performance of AI models varies across different ICH types and is more stable with larger sample sizes.

## 1. Introduction

Intracranial hemorrhage (ICH) is estimated to occur in approximately 30 out of 100,000 people annually [1]. Although ICH accounts for only 10–15% of all strokes, it contributes to more than half of all stroke cases [2]. Even if the patients survive, more than one-third suffer from severe disability [3]. In general, the early diagnosis of ICH is crucial as emergent surgery may be needed to save the patient’s life [4]. Depending on the anatomical location, ICH can be divided into intraparenchymal hemorrhage (IPH), subarachnoid hemorrhage (SAH), subdural hemorrhage (SDH), and epidural hemorrhage (EDH) [5]. The 30-day mortality rate of IPH is approximately 35–52%, and half of the deaths occur within the first day, highlighting the necessity of early treatment [6,7]. The most common cause of SAH is trauma; however, in non-traumatic cases, the rupture of an intracranial aneurysm accounts for up to 85% of the cases, which require early surgical clipping or endovascular coiling to prevent rebleeding [6]. Despite the different ICH patterns, the early diagnosis of ICH is crucial for reducing mortality. The diagnosis of choice for ICH was brain computed tomography (CT) without contrast injection [6]. Although ICH can usually be easily diagnosed using brain CT, delayed or missed diagnoses still frequently occur in daily practice for several reasons, including subtle hemorrhage, confusion with calcifications or artifacts, and radiologist fatigue owing to the high workload demand [8].

To reduce the risk of misdiagnosis, peer review by another radiologist has been proposed; however, it is not widely adopted owing to the additional time required [8]. The introduction of deep learning-based artificial intelligence (AI) algorithms has proved promising for the interpretation of medical images [9]. Several methods based on deep convolutional neural networks (CNNs) have been developed to detect and classify ICHs automatically [8]. Traditionally, these CNN techniques use two-dimensional (2D) CT images to develop AI models, and each image is predefined by a radiologist [10]. This time-consuming process can be avoided because the three-dimensional (3D) version, known as 3DCNN, is considered to provide highly accurate results when parsing complex medical images, such as CT, medical resonance imaging, and X-rays [11]. Notably, a 3DCNN can capture 3D spatial structural information in images better than their 2D counterparts. As a result, the physical and biological characteristics of the images can be incorporated into AI models [12]. A large database is considered to be an important factor in the performance of AI models. However, obtaining a large database from a single site is extremely time-intensive. Using an existing online dataset is more efficient than using a large database to develop AI models; however, the diversity of images, owing to different sources, different scan settings, or poor quality of controls, may make the database noisier. Although a carefully curated ICH database is already available, the application of the AI models developed from the database to the data of a single site requires the use of transfer-learning techniques [13]. Some relevant issues in transfer learning, such as catastrophic forgetting and overly biased pretrained models, need to be overcome [14]. In addition, the development of combined models with image and non-image data from existing databases is usually not possible because clinical information is largely limited. Other AI techniques such as radiomics are also valuable for building predictive models. Radiomics is increasingly employed to extract quantitative image features from medical images, thereby enabling the creation of analyzable databases. The radiomics process typically involves the following steps: (a) image acquisition and reconstruction; (b) image preprocessing; (c) the identification of regions of interest; (d) feature extraction and quantification; (e) feature selection; and (f) the development of predictive and prognostic models using machine learning [15,16].

Therefore, using a small in-hospital database to develop AI models is important; such a database allows for controlled image quality, ensures identical imaging scan settings of the trained models and tested data, and provides access to abundant clinical information. Sample size is crucial for the performance of AI models and is a topic widely discussed in the literature; however, the discussion is usually focused on hundreds or thousands of samples [17,18,19]. Occasionally, the data of interest are difficult to collect quickly, and the number of researchers available to collect the data is limited. The issue of cost–benefit ratios with regard to sample size is very important because an increase in sample size does not always change the effect size and accuracy [17]. In this study, we aimed to assess, using a limited number of cases, the number of enrolled cases with brain CT images that can achieve acceptable performance using AI models in terms of ICH detection via the new 3DCNN technique; this information will be helpful in gauging the cost–benefit ratio. In addition, we aimed to clarify whether different hemorrhage types affected the number of cases that achieved acceptable performance in the AI models.

## 2. Methods

### 2.1. Participants

This retrospective study was approved by the Institutional Review Board of Mackay Memorial Hospital and adhered to the ethical standards set by the Institutional Research Committee and Declaration of Helsinki. The study included patients who presented with acute ICH in our emergency department between January 2020 and December 2022. All CT images were obtained using the same scanner and settings. The inclusion criteria required the participants to be at least 20 years of age. ICH cases were identified and classified by an experienced neuroradiologist into the following categories: (1) IPH with or without intraventricular hemorrhage (IVH), (2) SAH with or without IVH, (3) SDH, (4) EDH, and (5) mixed ICH types.

The exclusion criteria were notable imaging motion artifacts, metallic artifacts, postoperative changes in the skull or intracranial region, and combined ICH types. Owing to the extremely limited number of EDH cases, these patients were excluded from the study. We consecutively enrolled 50 patients each with IPH, SAH, and SDH. Owing to anatomical considerations, IVH occasionally coexists with IPH or SAH. Consequently, patients with IPH or SAH with IVH were excluded from this study. Additionally, we included 150 non-hemorrhagic cases as the control group. The detailed patient characteristics for each group are provided in Table S1.

### 2.2. Model Training

#### 2.2.1. Preprocessing

**Figure 1:**
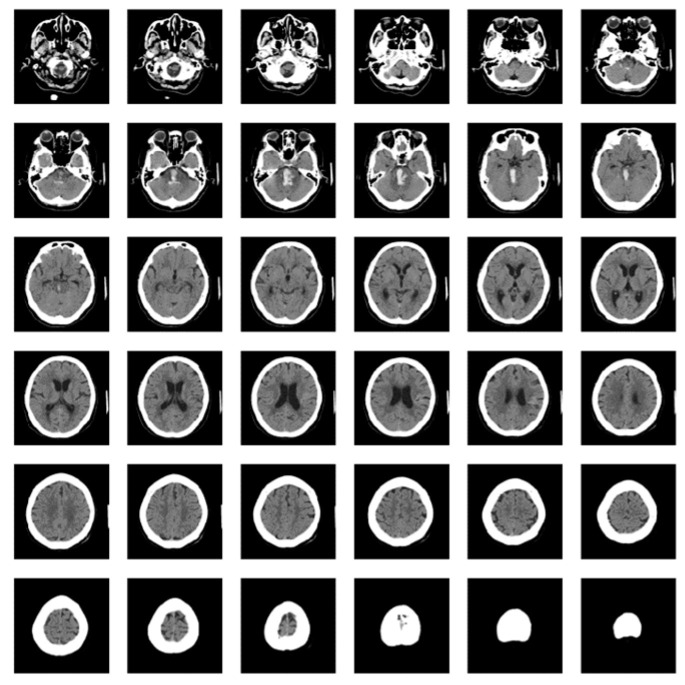
Images of one brain computed tomography dataset.

As shown in Figure 1, each brain CT scan consisted of 30–50 individual images combined to create 3D images. These 3D images were then used to train models to classify a patient depending on the presence or absence of an ICH.

Subsequently, we maintained the same model capacity, and randomly selected cases from a total of 150 non-hemorrhage patients and 50 patients with each hemorrhage type to train the models. We conducted a five-fold cross-validation to determine the specific number of cases needed for each hemorrhage type to achieve an area under the curve (AUC) exceeding 0.6. This process involved varying the numbers of non-hemorrhagic and ICH cases.

Models were developed using different combinations of non-hemorrhagic (ranging from 20 to 150) and ICH cases (ranging from 20 to 50). The dataset was divided into training and validation sets in a 4:1 ratio. For example, if 100 non-hemorrhagic and 40 ICH cases were used to develop the model, 80 non-hemorrhagic and 32 ICH cases were randomly selected for the training set, whereas the remaining 20 non-hemorrhagic and 8 ICH cases were used for validation. The details of the different case–number combinations used in this study are provided in Table 1.

After dividing the data into training and validation sets, the 2D CT images of each patient were stacked into 3D CT images. Each patient had 30–50 2D CT images, resulting in 3D CT images with heights corresponding to this range. To ensure consistency across input data, we adjusted the heights of all 3D CT images to a uniform value of 32. 

#### 2.2.2. 3DCNN

Traditional CNNs have been successful in image classification and recognition. The 3DCNN, an extension of the traditional 2DCNN model, excels in learning depth-related features from 3D images. This feature makes 3DCNNs particularly effective for medical imaging applications, in which depth and spatial context are crucial.

The CNN architecture includes convolutional and pooling layers for feature representation and fully connected layers for classification. The convolutional layer, which is a core component of a CNN, is primarily responsible for feature extraction. It utilizes convolution kernels that slide over the input images to capture various local features such as edges and textures. These filters are not manually predefined but learned and refined automatically during the training process of the model. By extracting crucial visual features from the input data, the convolutional layer provides valuable information to the subsequent layers of the network, thereby enhancing the recognition capabilities and overall performance of the model.

The pooling layer is primarily responsible for reducing the dimensionality of the data from the convolutional layer, while preserving important feature information. This process not only decreases the computational load of the model, but also helps mitigate overfitting. In our model, we used max pooling as the pooling operation. Max pooling selects the maximum value within a defined window on a feature map to represent a region. This approach effectively retains the most prominent features within a window and is less sensitive to noise.

The fully connected layer, positioned at the end of the CNN, functions similarly to the layers in traditional neural networks. It connects each input node to every output node, transforming the multidimensional feature information obtained from the convolution and pooling operations into one-dimensional data. The primary purpose of this layer is to integrate the local or regional features extracted by the previous layers into higher-order features. By providing this advanced feature representation, the fully connected layer enhances the ability of the model to accurately classify and identify the input data.

**Figure 2:**
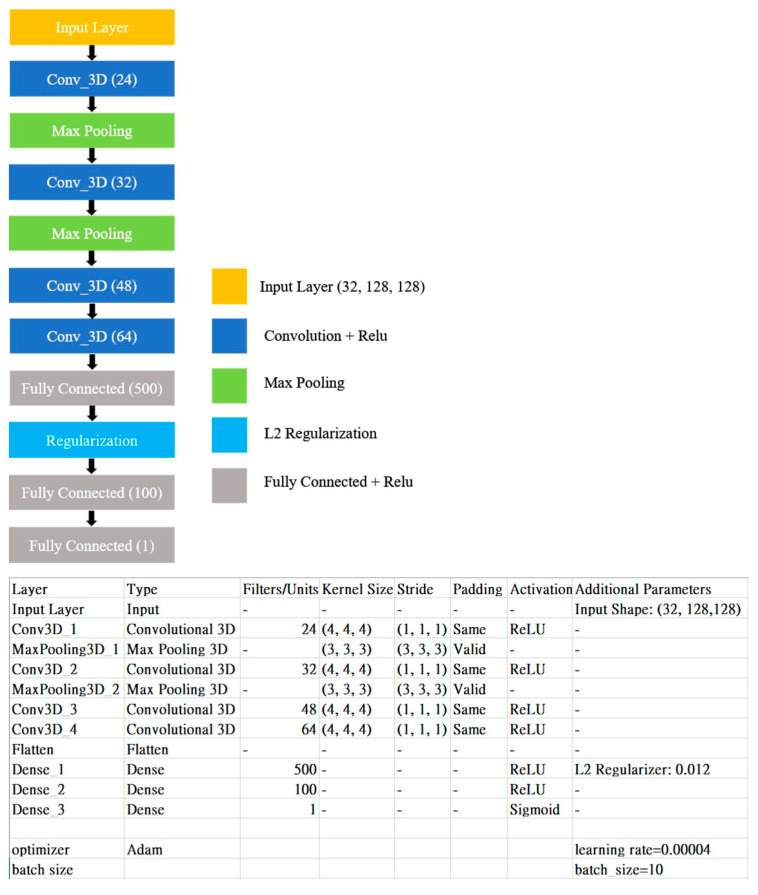
Three-dimensional convolutional neural network (3DCNN) structure.

The 3DCNN architecture used in this study is illustrated in Figure 2, with the relevant parameters given in each layer. The model begins with a preprocessed 3D image as the input, followed by feature extraction through four convolutional layers and two pooling layers. The classification is then performed using three fully connected layers. Regularization is incorporated into the fully connected layers to prevent model overfitting. The output from the final fully connected layer represents the result of the binary classification task with a sigmoid function as the activation function.

#### 2.2.3. AUC

The AUC is a key metric for evaluating model performance. It measures the area under the receiver operating characteristic (ROC) curve, which plots the false-positive rate on the x-axis against the true-positive rate on the y-axis. The AUC value ranges from 0.5 to 1.0, with a value closer to 1 indicating better model performance and classification accuracy. Conversely, an AUC value closer to 0.5 suggests poorer classification ability. In biomedical image classification, the AUC is a crucial indicator for assessing the effectiveness of a model.

#### 2.2.4. K-Fold Cross-Validation

K-fold cross validation involves dividing the data into K equal parts. The value of K can be selected based on the requirements. After splitting the data, (K-1) groups were used for training, whereas the remaining groups were reserved for validation. For example, in a five-fold cross-validation with 200 data points, 160 and 40 points were used for training and validation, respectively. This approach allows for averaging the results across different folds, minimizing errors during training. Cross-validation is especially beneficial with limited data because it helps reduce variance and improves the reliability of the results.

#### 2.2.5. Hyperparameter Tuning

The model contained 2,773,869 trainable parameters. Overfitting is a common problem encountered during training, particularly for small datasets. To address this issue, we use the “resize volume”, which refers to the number of images per case, should be controlled. Standardizing this parameter is crucial because of variations in the number of images per case. In addition, several hyperparameters, including the learning rate, batch size, kernel size, pool size, and L2 Regularization, can be adjusted. These hyperparameters (Figure 2) were manually fine-tuned to achieve optimal accuracy and AUC.

#### 2.2.6. External Validation

The external validation data used in this study were obtained from the CQ500 dataset ((accessed on 1 October 2024). https://arxiv.org/pdf/1803.05854). This dataset contained CT scans classified by three professional readers; only the scans with consistent classification results were used for validation. Target CT scans were selected based on our in-house criteria, including: (1) no hemorrhage, (2) pure SDH, (3) SAH with or without IVH, and (4) IPH with or without IVH. The final validation dataset included 245 non-hemorrhagic, 15 SDH, 9 SAH, and 53 IPH cases.

During model training, one-fifth of the cases were used for validation, resulting in validation set combinations of 4–30 non-hemorrhagic and 4–10 hemorrhagic cases. To align with these combinations, we selected the first 10 cases of each hemorrhage type and the first 30 non-hemorrhagic cases from CQ500. However, because of the presence of only 9 SAH cases, we included an additional non-hemorrhagic case to meet the requirement of 10 SAH cases. The performances of the models on in-house data and CQ500 were compared, with a difference of less than 0.1 in accuracy considered similar in this study.

## 3. Results

The enrolled 300 participants included 170 men and 130 women, with a mean age of 62.35 ± 17.09 years (ranging from 20 to 98 years). This study aimed to investigate changes in AUC and accuracy based on varying non-hemorrhagic-to-ICH case ratios. Different hemorrhage types exhibited distinct fluctuations in these ratios.

In this study, we recorded three different AUC values: the Average AUC of the Last Five Epochs (representing the average AUC of the last five generations of training), the Average Last Epoch Validation AUC (representing the average AUC of the last generation of training), and the Average Validation AUC (representing the AUC for each generation of training). These average AUC values for the SDH hemorrhage type are presented in Table 2, and the values were obtained based on the data obtained from 50 ICH cases in the SDH type and various non-hemorrhagic cases (ranging from 20 to 150). Upon observation, it was noticed that the Average Validation AUC showed slightly lower values than the other metrics. To assess the impact of sample size on the AUC, we relied on the more stable Average AUC of the Last Five Epochs. Although the Average Last Epoch Validation AUC closely resembled the Average AUC of the Last Five Epochs, only the AUC of the last generation could have led to a biased recording of high and low values.

**Figure 3:**
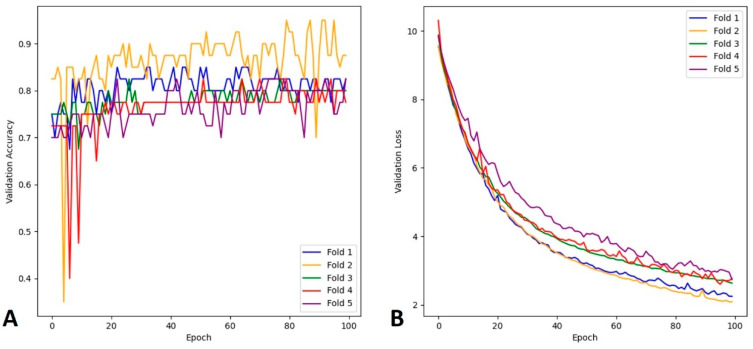
Subarachnoid hemorrhage (SAH) data with 150 non-hemorrhagic and 50 hemorrhagic cases: (**A**) accuracy, (**B**) loss.

In this study, we implemented a five-fold cross-validation, and randomly selected patients with ICH and without hemorrhage for binary classification training. For instance, if we chose 150 non-hemorrhagic and 50 ICH patients, we would have 120 non-hemorrhagic and 40 ICH training samples. The accuracy curve for the ICH training data demonstrated using the SAH cases is presented in Figure 3A. The accuracy curves presented in the figure correspond to each training epoch, with a total of five curves. Each set of training results is associated with a set of loss curves, as shown in Figure 3B.

**Figure 4:**
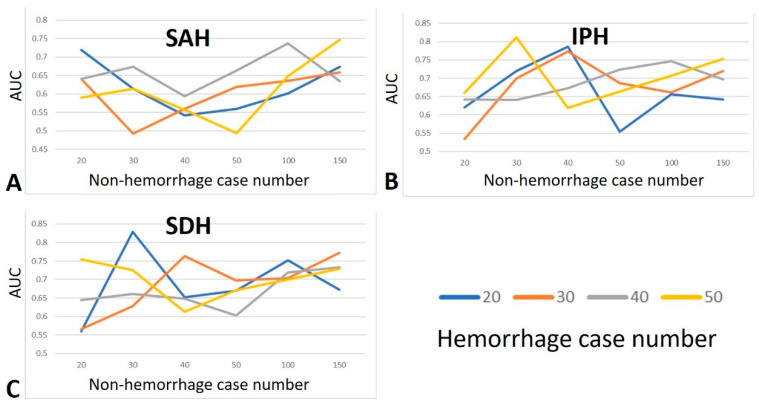
Area under the curve (AUC) values for the (**A**) SAH, (**B**) IPH, and (**C**) SDH types.

The SAH type, with a very small sample size, was prone to yield excessively high AUC. As the number of non-hemorrhagic cases increased, the overall AUC initially decreased, before subsequently increasing to a peak when the number of non-hemorrhagic cases exceeded 100 (Figure 4A). In the case of IPH, the AUC values fluctuated considerably before the 50 non-hemorrhagic cases, irrespective of the number of hemorrhagic cases. After 50 non-hemorrhagic cases, the overall AUC values showed a trend of gradually increasing to 150 non-hemorrhagic cases (Figure 4B). In the case of the SDH type, the overall patterns of the AUC values were similar to those of the IPH type, showing marked fluctuations before 50 non-hemorrhagic cases, followed by a gradual increase to 150 non-hemorrhagic cases (Figure 4C).

**Figure 5:**
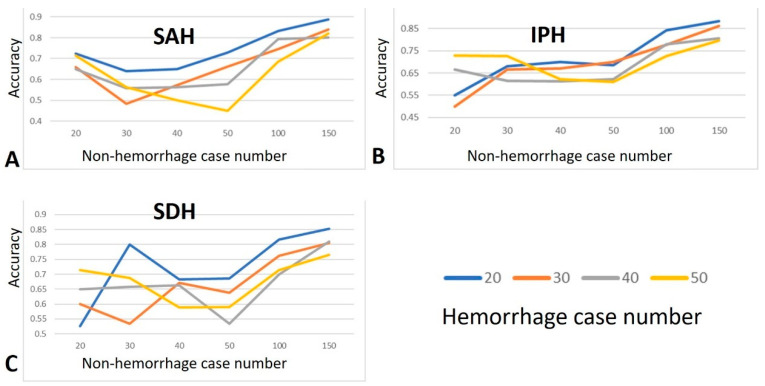
Accuracy values for the (**A**) SAH, (**B**) IPH, and (**C**) SDH types.

Irrespective of the bleeding type, the accuracy of the data was heavily influenced by the volume of data available. Consequently, an increase in the overall data volume led to a notable improvement in the accuracy, as shown in Figure 5. However, when the quantity of the ICH data was limited, the accuracy tended to increase. This falsely high accuracy resulted from the consistent use of a 4:1 training-to-validation ratio during the cross-validation. A higher accuracy rate is more likely to be achieved when the data pool is small.

The accuracy of the model’s performances between the in-house and external validation (CQ500 data) data is detailed in Table 3, Table 4 and Table 5. A total of 38 out of the 72 conditions showed a similar accuracy of less than 0.1. Three conditions showed better performance in the external validation than in the in-house data, whereas the remaining 31 conditions showed better performance in the in-house data. In the SAH, there were 14 conditions with similar model performances, 8 with better performances in the in-house data, and 2 with better performances in the external validation data. In IPH, there were 8 conditions with similar model performances, 15 with better performances in the in-house data, and 1 with better performance in the external validation data. In SDH, there were 16 conditions with similar model performances and 8 with better performances in the in-house data.

## 4. Discussion

In our study, datasets with a higher number of enrolled cases were more likely to yield a stable and acceptable AI model performance, particularly in terms of AUC and accuracy, with the trend being more pronounced for accuracy than for AUC. Training AI models with a limited number of cases sometimes results in deceptively high AUC or accuracy values, whereas larger datasets produce more stable and consistent results. While the AUC trends and fluctuations across different ICH types varied depending on the case ratios, the accuracy trends remained relatively similar across different ICH types.

In a limited dataset, including more cases is important for achieving a stable and acceptable AI model performance, particularly in terms of AUC, with the effect being even more pronounced for accuracy than for AUC. In this study, we trained the AI models for each hemorrhage type using varying numbers of non-hemorrhagic (20–150) and ICH cases (20–50). For the SAH type, the AUC values exhibited a U-shaped trend, with relatively high values observed for both small and large numbers of non-hemorrhagic cases. In contrast, for IPH, the AUC values fluctuated considerably, with fewer than 50 non-hemorrhagic cases. A similar pattern was observed for the SDH type, where the AUC values fluctuated markedly before reaching 50 non-hemorrhagic cases. To achieve an AUC value greater than 0.6, at least 100 non-hemorrhagic and 20 ICH cases are required for both the SAH and IPH types, whereas the SDH type only requires 30 non-hemorrhagic cases. If an AI model aims to identify all three ICH conditions with an AUC value of over 0.6, enrolling at least 20 cases for each ICH type and at least 100 non-hemorrhagic cases is recommended. However, to achieve an AUC value greater than 0.7, our study found that only the condition with 50 ICH cases and 150 non-hemorrhagic cases reached this threshold for all three ICH types. Using this information, researchers can estimate the necessary sample size based on the desired AUC values. In addition, the specific ICH type studied influenced the required number of cases. The accuracy results were more consistent across the three ICH types, showing a gradual increase after 50 non-hemorrhagic cases and reaching an accuracy above 0.7 when the non-hemorrhagic case count exceeded 100. Although the condition with 20 ICH cases showed the highest accuracy, this was likely due to a false accuracy phenomenon caused by the limitations of a very small sample size. Increasing the non-hemorrhagic case count to 150 further improved the accuracy, and the results became more similar across different ICH case numbers. To achieve accuracy between 0.75 and 0.85 in ICH prediction, conditions with 150 non-hemorrhagic and either 40 or 50 ICH cases met this goal for all three ICH types. Therefore, we suggest that at least 150 non-hemorrhagic and 40 cases each of the ICH types are necessary to develop an AI model with stable and high accuracy. Notably, the conditions with very limited case numbers sometimes produce very high accuracy, which should be carefully scrutinized because it may reflect false accuracy due to the inherent limitations of small sample sizes.

When training AI models with a limited number of cases, there is a risk of obtaining inflated AUC values and accuracy. In contrast, using a larger case count, even with varying non-hemorrhage-to-ICH ratios, tends to produce more consistent results. In this study, the trend of AUC values for the SAH type showed comparable results between the non-hemorrhage case counts of 20 and 150. However, considerable fluctuations in the AUC values were observed for the IPH and SDH types when the non-hemorrhagic case count was less than 50. In the SAH type, the accuracy remained relatively high even with 20 non-hemorrhagic cases, and in the SDH type, the accuracy reached 0.8, with 30 non-hemorrhagic and 20 ICH cases. Accuracy is highly sensitive to the sample size, with smaller datasets showing much wider variations than larger ones [20]. The risk of sampling errors in small datasets can result in an unreasonably high or low accuracy. Previous reviews have highlighted the negative correlation between sample size and accuracy in similar research fields [18,21], suggesting that results from studies with small sample sizes should be interpreted with caution. Unlike the accuracy, which is calculated at a single operating point, the AUC considers all operating points on an ROC curve [22]. Thus, the AUC is a more reliable and preferred metric than the accuracy for binary classification. However, the AUC is also subject to greater variability than the accuracy in small datasets [19]. Because the AUC represents balanced average accuracy, errors in the minority class are assigned equal weights to those in the majority class, which can exaggerate the impact of small sample sizes [22]. Therefore, both the AUC and accuracy should be interpreted carefully when dealing with small datasets.

The AUC results appeared to vary more across ICH types, whereas the accuracy values were more consistent. In this study, the accuracy outcomes across the three ICH types were more uniform than the AUC, possibly due to the relatively simple calculation for accuracy. The variation in AUC within each ICH type was greater than that in accuracy, and the overall trends among the ICH types were different. However, with the increase in the sample size, the variations and differences became less notable. While the specific ICH type being studied may influence the AI model’s performance, this effect can be minimized with larger sample sizes, as evidenced by studies involving extremely large datasets [8].

The total sample size in this study was relatively small, with the combined number of non-hemorrhagic and hemorrhage cases ranging from 40 to 200 for developing the AI models. The definition of a small sample size varies across studies, with some defining it as fewer than 100 cases [18]. Using a small sample size to create AI models can lead to several challenges, such as inflated accuracy owing to overfitting or random effects [17], which was observed in our results when the number of non-hemorrhagic cases was less than 50. Another issue with small sample sizes is that model performance can be substantially influenced by the choice of analytical method [23]. In addition to the 3DCNN, we experimented with Long Short-Term Memory (LSTM), a Gated Recurrent Unit (GRU), and a combination of LSTM and GRU. However, none of these methods produced models with an accuracy above 0.7 in any condition, generally performing worse than the 3DCNN-based models. Besides this, no universally accepted threshold exists for distinguishing between small and large sample sizes; however, the concept of “appropriate” sample size is more practical. The appropriate size can vary depending on the research topic, data characteristics, and machine learning methods used [17]. Both the sample and effect sizes considerably affected the performance of the AI models. When the effect size is large, data classification becomes easier, allowing many machine learning methods to produce excellent models even with a small sample size. However, some methods require a larger sample size to achieve good performance. Conversely, when the effect size is small, classification becomes more difficult, and the model performance can vary substantially depending on the machine learning method used, especially with a small sample size [17]. In such cases, exploring different machine learning methods can be beneficial if the performance of the initial model is unsatisfactory, particularly when both the sample and effect sizes are small.

Class imbalance is an important issue in machine learning, particularly when datasets are highly imbalanced. In such cases, classifiers tend to prioritize maximizing the overall predictive accuracy by focusing on the majority class, potentially leading to the misclassification or neglect of the minority class. In the medical field, this problem is particularly concerning because disease conditions are typically far less common than normal conditions. Consequently, classifiers trained on imbalanced datasets may struggle to detect rare but crucial disease conditions [24]. In our study, we observed that as the number of non-hemorrhagic cases increased, the overall accuracy improved across all three hemorrhage types. However, this increase was likely driven by the enhanced performance in identifying the majority of non-hemorrhagic cases, while the detection of minority hemorrhagic cases remained stable or even declined. Several techniques can be employed to address class imbalance, such as undersampling the majority class, oversampling the minority class, and modifying the classification algorithms to assign more weight to the minority class. However, these methods have potential drawbacks, including the risk of discarding valuable data, overfitting the model, and extended training times [24,25]. Although these techniques can be helpful, their limitations must be carefully considered when they are applied in practice.

In the external validation process, 31 of the 72 conditions showed the worse performance of our models on the external data, 3 showed better performance, and 38 demonstrated similar performances. This result indicates that our models exhibited remarkably worse performance in over 40% of the conditions when applied to external datasets, highlighting generalization issues, particularly with models developed from a small sample size. While increasing the sample size could improve the performance during external validation, incorporating more diverse data is also important in addressing this problem [26]. Approximately 60% of the conditions in the SAH and SDH types maintained similar accuracies in the external validation, whereas more than 60% of the conditions in the IPH type showed a decline in performance. This finding suggests that the ability of a model to generalize is influenced not only by the sample size but also by the specific classification target. Another potential confounding factor was the total hemorrhage volume, which could have influenced the evaluation. While it is well established that sample size plays a crucial role in improving generalization, our study suggests that the nature of target classification, which is potentially influenced by the effect size of the data, may also be an important factor. However, further research is needed to investigate how sample size and target characteristics interact, and their combined impact on the generalization of AI models.

This study has several limitations. First, the number of cases was limited, and as a result, the performance of the developed AI models was expected to be suboptimal. However, we intentionally constrained the number of cases because our goal was to develop AI models from a limited dataset, which reflects the practical constraints faced by many research centers with limited resources. Our study demonstrates that it is feasible to develop AI models for ICH detection with acceptable performance using a small sample size. Although this approach can be extended to other medical conditions, further research is required to validate these findings. Additionally, all images used in this study were acquired using the same CT scanner with identical scan settings, which raises concerns regarding the generalization of our results to imaging data from other scanners. However, the use of a uniform dataset may have resulted in an improved model performance owing to the reduced variation in image quality. Another limitation is that we used only the basic 3DCNN architecture. Future work could explore the integration of transfer learning to improve model performance. Transfer learning enables models to leverage knowledge from large datasets and apply it to smaller specific datasets, thereby potentially enhancing their performance in specialized classification tasks. Lastly, the issue of sample size is particularly important when working with 3D data, as it is subject to the “curse of dimensionality”. This principle suggests that the sample size required for optimal model performance increases exponentially with higher-dimensional data, such as 3D imaging, compared with lower-dimensional 2D data. Therefore, carefully addressing the sample size is crucial in studies involving 3D data to ensure a robust model development. Furthermore, the performance of the AI models in detecting ICH may be affected by the size and density of the hemorrhage. In this study, we did not impose constraints on ICH grade; however, it would be interesting to investigate the detection rate within a more homogeneous ICH group characterized by a consistent hemorrhage size and density in future research.

## 5. Conclusions

The 3DCNN technique can be used to develop AI models with acceptable performance in the detection of ICH from a limited number of cases; however, there is a minimum required case number suggested by this study to decrease the variation in the result and increase the performance of the models. The performance of the AI models varies for different ICH types; however, the differences become less obvious when a larger sample size is used.

## Figures and Tables

**Table 1:** Training and validation sample distribution.

	Non-hemorrhage					
Enrolled case numbers	**20**	**30**	**40**	**50**	**100**	**150**
Training:Validation	16:4	24:6	32:8	40:10	80:20	120:30
	Intracerebral hemorrhage					
Enrolled case numbers	**20**	**30**		**40**		**50**
Training:Validation	16:4	24:6		32:8		40:10

**Table 2:** Area under the curve (AUC) values for subdural hemorrhage (SDH).

SDH	20	30	40	50	100	150
**Average AUC of Last Five Epochs**	0.6602	0.8119	0.6189	0.6638	0.7071	0.7536
**Average last_epoch_val_AUC**	0.6652	0.8100	0.6166	0.6635	0.7033	0.7595
**Average Validation AUC**	0.6518	0.7859	0.6104	0.6406	0.6818	0.7188

**Table 3:** Accuracy of model performance using the in-house and external validation (CQ500 data) data for subarachnoid hemorrhage (SAH).

Accuracy In-House/CQ500		Non-Hemorrhagic Case					
		20	30	40	50	100	150
SAH Hemorrhagic case	20	0.73/0.63	0.64/0.40	0.65/0.33	**0.73/0.71**	**0.83/0.83**	**0.89/0.88**
	30	**0.66/0.60**	0.48/0.58	0.57/0.40	**0.66/0.63**	**0.75/0.77**	**0.84/0.78**
	40	**0.65/0.67**	**0.56/0.57**	**0.56/0.50**	0.58/0.44	0.79/0.46	**0.80/0.79**
	50	**0.71/0.71**	**0.56/0.63**	**0.50/0.55**	0.45/0.65	0.69/0.33	0.82/0.25

Bold-faced texts indicate a difference of accuracy less than 0.10. Underlined texts indicate better performance in the external validation data, while regular texts indicate better performance in the in-house data.

**Table 4:** Accuracy of model performance in the in-house and external validation (CQ500 data) data for intraparenchymal hemorrhage (IPH).

Accuracy In-House/CQ500		Non-Hemorrhagic Case					
		20	30	40	50	100	150
IPH Hemorrhagic case	20	**0.55/0.63**	0.68/0.40	0.70/0.17	0.69/0.57	**0.84/0.83**	0.88/0.18
	30	0.50/0.60	0.67/0.50	0.67/0.50	0.70/0.31	0.78/0.23	**0.86/0.83**
	40	**0.67/0.67**	**0.61/0.57**	0.61/0.50	**0.62/0.55**	0.78/0.29	0.81/0.58
	50	**0.73/0.64**	0.73/0.63	**0.62/0.55**	0.61/0.45	0.73/0.33	0.80/0.25

Bold-faced texts indicate a difference of accuracy less than 0.10. Underlined texts indicate better performance in the external validation date, while regular texts indicate better performance in the in-house data.

**Table 5:** Accuracy of model performance in the in-house and external validation (CQ500 data) data for SDH.

Accuracy In-House/CQ500		Non-Hemorrhagic Case					
		20	30	40	50	100	150
SDH Hemorrhagic case	20	**0.53/0.50**	0.80/0.30	**0.68/0.67**	**0.69/0.71**	**0.82/0.83**	**0.85/0.88**
	30	0.60/0.50	**0.53/0.50**	0.67/0.50	**0.64/0.63**	**0.76/0.77**	**0.81/0.83**
	40	**0.65/0.66**	0.66/0.50	0.66/0.38	**0.53/0.56**	**0.70/0.71**	**0.81/0.79**
	50	0.71/0.29	**0.69/0.63**	0.59/0.44	0.59/0.45	**0.71/0.67**	**0.76/0.77**

Bold text indicating a difference of accuracy of less than 0.10. Underlying text indicating better performance in external validation and regular text indicating better performance in in-house data.

## Data Availability

The datasets used in this study are not publicly available because of ethical restrictions preventing the public sharing of data.

## Supplementary Materials

The following supporting information can be downloaded from: https://www.mdpi.com/article/10.3390/diagnostics15020216/s1. Table S1: Patient characteristics of the dataset.
