# Supplementary material for: Impact of Dataset Size on 3D CNN Performance in Intracranial Hemorrhage Classification
Source: Diagnostics (Basel). 2025 Jan 18;15(2):216. doi: 10.3390/diagnostics15020216 (PMC11763925; doi:10.3390/diagnostics15020216)
Supplement: Supplementary file 1 [file diagnostics-15-00216-s001.zip › diagnostics-3337652-supplementary.pdf]

	Non-hemorrhage	SAH	IPH	SDH
Case number	150	50	50	50
Sex (Male, %)	85 (56.7)	23 (46.0)	32 (64.0)	30 (60.0)
Age (Mean $\pm$ SD) [Range]	59.5 $\pm$ 18.4 [21-98]	59.5 $\pm$ 16.3 [20-92]	62.4 $\pm$ 12.5 [38-92]	73.7 $\pm$ 13.0 [41-96]

Supplementary Table S1. Patient characteristics of the dataset. Abbreviations: IPH, intraparenchymal hemorrhage; SAH, subarachnoid hemorrhage; SD, standard deviation; SDH, subdural hemorrhage
